# Smooth Muscle miRNAs Are Critical for Post-Natal Regulation of Blood Pressure and Vascular Function

**DOI:** 10.1371/journal.pone.0018869

**Published:** 2011-04-22

**Authors:** Sebastian Albinsson, Athanasia Skoura, Jun Yu, Annarita DiLorenzo, Carlos Fernández-Hernando, Stefan Offermanns, Joseph M. Miano, William C. Sessa

**Affiliations:** 1 Department of Pharmacology, Vascular Biology and Therapeutics Program, Yale University School of Medicine, New Haven, Connecticut, United States of America; 2 Max-Planck-Institute for Heart and Lung Research, Bad Nauheim, Germany; 3 Aab Cardiovascular Research Institute, University of Rochester School of Medicine and Dentistry, Rochester, New York, United States of America; University College London United Kingdom

## Abstract

Phenotypic modulation of smooth muscle cells (SMCs) plays a key role in vascular disease, including atherosclerosis. Several transcription factors have been suggested to regulate phenotypic modulation of SMCs but the decisive mechanisms remain unknown. Recent reports suggest that specific microRNAs (miRNAs) are involved in SMC differentiation and vascular disease but the global role of miRNAs in postnatal vascular SMC has not been elucidated. Thus, the objective of this study was to identify the role of Dicer-dependent miRNAs for blood pressure regulation and vascular SMC contractile function and differentiation *in vivo*. Tamoxifen-inducible and SMC specific deletion of Dicer was achieved by Cre-Lox recombination. Deletion of Dicer resulted in a global loss of miRNAs in aortic SMC. Furthermore, Dicer-deficient mice exhibited a dramatic reduction in blood pressure due to significant loss of vascular contractile function and SMC contractile differentiation as well as vascular remodeling. Several of these results are consistent with our previous observations in SM-Dicer deficient embryos. Therefore, miRNAs are essential for maintaining blood pressure and contractile function in resistance vessels. Although the phenotype of miR-143/145 deficient mice resembles the loss of Dicer, the phenotypes of SM-Dicer KO mice were far more severe suggesting that additional miRNAs are involved in maintaining postnatal SMC differentiation.

## Introduction

*In vivo*, medial vascular smooth muscle cells (VSMCs) are normally quiescent and programmed for contraction but under certain conditions these cells undergo phenotypic modulation which may result in increased proliferation and/or migration from the media [1]. Phenotypic modulation of VSMCs is likely a key mechanism to allow repair of vascular injury but is also recognized as one important event in multi-factorial vascular disease states such as arteriosclerosis, re-stenosis and vein graft occlusion [1]. In addition, remodeling and hypertrophy of VSMCs may be involved in the progression of essential hypertension.

The mechanisms of VSMC phenotypic modulation remain elusive despite intense investigation. However, several transcription factors, including serum response factor (SRF), myocardin, myocardin related transcription factors (MRTFs) and members of the Krüppel-like zinc finger family (KLF) have been suggested to act as molecular switches regulating VSMC differentiation [2], [3]. During the last decade, microRNAs (miRNAs), which are short (∼22 nt) noncoding RNAs have been suggested to regulate mRNA expression levels and translational efficiency and play a fundamental role in a number of human disease states, including vascular disease [4], [5]. Specific miRNAs have been shown to regulate VSMC phenotypic modulation and/or control VSMC fate and differentiation [6]–[12]. miR-21 and miR-221 were initially found to play a role in SMC proliferation and differentiation [7]–[10] and more recently, miR-145 was shown to be specifically expressed in SMCs and play an important role in SMC differentiation [6], [11], [13], [14]. Studies on miR-143/145 KO mice later revealed that these miRNAs are important but not essential for VSMC development *in vivo* [13]–[15].

miRNAs are produced from immature pre-miRNAs, which are processed by the two RNase III endonucleases, Drosha and Dicer, and are then incorporated into the RNA-induced silencing complex (RISC) [16]. Depending on the complementarity of the miRNA with the 3'-untranslated region of the target mRNA, the RISC complex will mediate either translational repression/activation or degradation of the target mRNA. Since Dicer is required for processing of almost all mature miRNAs, mutation or disruption of Dicer has been widely used as an approach to investigate the biological significance of miRNAs in various cell types including cardiomyocytes [17]–[19], endothelial cells [20]–[22], fibroblasts [23] and immune cells 24–26]. Our group recently reported that conditional loss of Dicer in VSMCs during embryonic development results in embryonic lethality associated with extensive internal hemorrhage as well as dilated and thin walled blood vessels caused by a reduction in cellular proliferation [27]. In addition, arteries from SM-Dicer KO embryos exhibited impaired contractility due to a loss of contractile differentiation of VSMCs. In isolated Dicer KO VSMCs, loss of contractile differentiation was rescued by miR-145 mimic, possibly via increased actin polymerization. Although miR-145 can rescue several defects in cultured SMCs, the absence of a lethal phenotype of miR-145 KO mice suggests that other miRNAs or combinations of miRNAs are important for SMC development and function.

Since deletion of Dicer during prenatal development is embryonic lethal it is not possible to investigate the importance of miRNAs in VSMC function and blood pressure regulation in adult mice using this model. In order to address this question we used a newly developed tamoxifen-inducible and SMC specific Cre mouse line [28] bred to a well established Dicer^floxed^ mouse line [24]. Here we show that the loss of miRNAs in SMC in adult mice results in a dramatic decrease in blood pressure due to loss of contractile function, phenotypic modulation of SMC and vascular remodelling. These data indicate that active turnover of mIRNA is critical to maintain post-natal differentiation of vascular smooth muscle in blood vessels.

## Methods

### Animals

Adult mice with inducible and SMC specific inactivation of Dicer were generated by intercrossing mice harboring loxP sites flanking an RNaseIII domain (exons 20 and 21) of Dicer [24] with transgenic mice expressing the tamoxifen-dependent Cre recombinase CreER^T2^ under control of the SMC-specific myosin heavy chain (SM-MHC) promoter [28]. At the age of 3–4 weeks, male SMMHC-CreER^T2^/Dicer^flox/flox^ (SM-Dicer KO) mice were treated with intraperitoneal injections of approximately 0.1 ml Tamoxifen (50 mg/kg/day) or vehicle (1∶10 EtOH in sunflower oil) for 5 consecutive days. Vehicle treated littermate mice were used as controls. In several experiments, male, Cre negative, Tamoxifen and vehicle treated mice were used as additional controls for any effect of Tamoxifen. For most experiments, mice were used either 4–5 weeks or 8–10 weeks post tamoxifen treatment. All mice were on a mixed C57Bl/6;129 background and all animal procedures were approved by the Yale Animal Care Committee (protocol 07919).

### Blood pressure measurement

Blood pressure in conscious male mice (6–8 weeks post Tamoxifen treatment) was non-invasively measured by determining the tail blood volume with a volume pressure recording sensor and an occlusion tail-cuff (CODA system, Kent Scientific Corporation, Torrington, CT). Mice were placed in warmed restraining chambers and acclimatized to the experimental procedure for a week before the data acquisition. After first five data points were discarded, readings were recorded for 25 cycles. Data points for each animal were collected to determine heart rate, systolic and diastolic blood pressure.

### Echocardiography studies

The cardiac dimensions of conscious mice were monitored using transthoracic echocardiography (Vevo 770 VisualSonics, Toronto, Canada), both before and after the 8 weeks of treatment as described [29]. In brief, left ventricular (LV) M-mode (VisualSonics) was used and all measurements were made during 3 to 6 consecutive cardiac cycles, and the average values were used for analysis. LV end-diastolic (LVDd) and end-systolic (LVDs) dimensions, as well as the thickness of the interventricular septum (IVST) and posterior wall (PWT) were measured from the M-mode tracings, and fractional shortening (FS) was calculated as (LVDd-LVDs)/LVDd. Diastolic measurements were taken at the point of maximum cavity, and systolic measurements were made at the point of minimum dimension, using the leading-edge method.

### Force measurements

Male mice were sacrificed either 5 or 10 weeks following Tamoxifen injections. Mouse saphenous and second order mesenteric arteries were isolated, dissected free from surrounding tissue and mounted in a myograph (610M; Danish MyoTechnology, Aarhus, Denmark). Following 1h equilibration in HEPES-buffered Krebs solution (composition in mM: 135.5 NaCl, 5.9 KCl, 2.5 mM Ca^2+^, 1.2 MgCl2, 11.6 glucose, 11.6 HEPES, pH 7.4) at a preload of 2.5 mN, vessels were contracted twice using High-KCl HEPES-buffer (HK) obtained by exchanging 80 mM NaCl for KCl. Following 7 min of contraction in HK-buffer vessels were relaxed by washout using HEPES-buffered Krebs solution and the remaining tone was measured after 1 minute. Saphenous arteries were then subjected to cumulative dose-response relationships to phenylephrine (PE) and serotonin (5-HT) Active and passive circumference-tension relationships were generated in mesenteric arteries as described previously [27].

### Vessel morphometry and SMC number

Upon anesthesia, vessels from control and SM-Dicer KO mice 10 weeks post tamoxifen treatment were dilated using sodium nitroprusside and adenosine, perfusion fixed in 4% PFA in PBS, pH 7.4 and embedded into O.C.T compound (Tissue-Tek, Sakura Finetek USA, Inc). H&E staining was performed on 4 independent cross-sections (7 µm) from 4–5 animals per each group. Images were captured using Nikon Eclipse 80i and morphometric analysis was performed using NIS Elements Imaging Software (Nikon Instruments Inc.). Circumference measurements of the external elastic lamina (EEL) and the internal elastic lamina (IEL) were used to calculate the area and vessel thickness.

For immunofluorescence, sections of vessels from Ctrl and SM-Dicer KO mice were mounted on glass slides and stained using a fluorescently labeled SM α-actin antibody (Sigma) and DAPI nucleic acid stain. SMC cell number in the aortic media was analyzed in 12–15 sections from 4–5 animals.

### Transmission Electron Microscopy

Aorta and saphenous artery from Control or SM-Dicer KO Dicer mice (n = 3 for each condition) were fixed in 0.1M phosphate buffered 2.5% glutaraldehyde and processed for ultrastructural analysis using a Hitachi 7650 transmission electron microscope as described previously [27]. All samples were coded for an unbiased assessment of the general organization of the vessel wall and medial SMC ultrastructure. Initial images were captured with a GATAN Erlangshen 11 mega pixel digital camera and processed in final format with Adobe Photoshop.

### Calcium measurements

Isolated, primary aortic SMCs were incubated in HEPES buffered Krebs solution containing 2,5 µM Fluo-4 AM (Molecular Probes) and 0.02% Pluronic F-127 (Invitrogen) for 30 min at room temperature in the dark. The loading solution was replaced with fresh buffer and the cells incubated for an additional 30 min to allow de-esterification of the dye. Baseline fluorescence was acquired for 30 seconds prior to addition of any agonist with one image acquired every 3 seconds. Following addition of the agonist images were acquired for an additional 100 seconds. All cells within the field of view were analyzed separately using ImageJ and the average of these cells was calculated as a single value for each time point. Separate isolations from 3 animals were tested in duplicates.

### Quantitative real time PCR (qPCR)

qPCR for *Dicer* and selected miRNAs was performed as reported previously [21], [22]. Briefly total RNA was extracted from intact aortae including the adventitia, media and intima using Qiagen miRNeasy mini kit according to the manufacturer's instructions. Reverse transcription was performed using Taqman Reverse Transcription Reagents (Applied Biosystems) for mRNA and RT2 miRNA First Strand kit for miRNAs. The qPCR primer sequences used for *Acta2, Cnn1, Myh11, Myocd and Kcnmb1* have been published previously [30]. Commercially available primer sequences for selected miRNAs were used (SA Biosciences). 18S RNA and sno142 were used as house-keeping genes for mRNA and miRNA respectively. qPCR reactions were analyzed on BioRad IQ5 (BioRad Laboratories).

### Immunoblotting

Standard Western blot analysis was conducted using Dicer (Santa Cruz Biotechnology Inc.), HSP90 (BD Biosciences), α-actin (Sigma), SM-MHC (Biomedical Technologies Inc.), Calponin (Abcam) and SM22 (Abcam) antibodies.

### Statistical analysis

Graphs and statistical analysis were prepared using GraphPad Prism (GraphPad Software) All data are presented as mean ± SEM and single comparison between two groups was performed using student's t-test. Multiple comparisons were performed using ANOVA and Bonferroni post test. Two-way ANOVA was used for statistical analysis of differences of curves such as dose response relationships and statistical significant differences between genotypes in these cases are indicated for all data points collectively.

## Results

### Mice with inducible smooth muscle specific deletion of Dicer exhibit near complete loss of smooth muscle specific miR-145

Dicer is essential for the maturation of the majority of miRNAs and deletion of Dicer therefore subsequently results in a loss of these miRNAs. Mice homozygous for Dicer flanked by LoxP sites [24] were bred with an inducible, SMC specific Cre transgenic mouse line [28] in order to generate a SMC specific knock down of Dicer. Cre expression in this mouse line has previously been demonstrated to be specific for vascular and visceral SMCs [28]. Figure S1A (lower panel), shows efficient excision of the floxed Dicer allele in genomic DNA from tail of Cre-positive mice 2 weeks post tamoxifen treatment. Conventional PCR was only used for genotyping of the mice prior to experiments since it was not possible to quantify the level of Dicer knockdown using this method.

**Figure 1:**
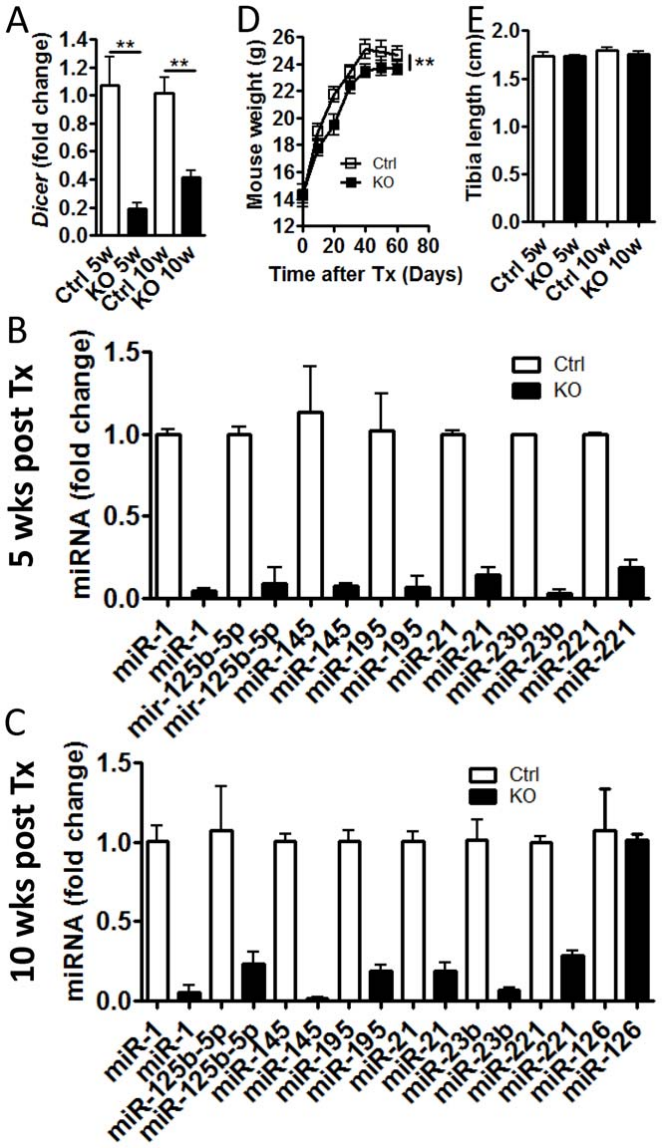
Deletion of SM-Dicer results in a reduction in miRNA expression and weight loss. Dicer mRNA (A) and selected miRNAs (B and C) were analyzed using RT-qPCR in intact aortas from control (Ctrl) and SM-Dicer KO (KO) mice, 5 or 10 weeks post tamoxifen. Data are mean +SEM, n = 3–6 aortae per group. (D) Mouse weight was measured every 10 days for 9 weeks after tamoxifen treatment in control (Ctrl) and SM-Dicer KO (KO) mice (n = 9–26). (E) Tibia length was measured in KO and Ctrl mice at 5 or 10 weeks post tamoxifen treatment (n = 3–8).

To confirm loss of Dicer and miRNAs in VSMC, Dicer mRNA and several miRNAs were analyzed by RT-qPCR in control and SM-Dicer KO aorta. The reduction in Dicer mRNA levels 5 weeks post tamoxifen exceeded 80% (Figure 1A). At 10 weeks post tamoxifen, the level of Dicer was reduced by approximately 60%. We have previously shown that cultured aortic VSMC isolated from SM-Dicer KO mice 10 weeks post-tamoxifen have a >80% knock down of Dicer [27]. Of note, at 5 and 10 weeks, the SMC enriched miRNA, miR-145, was reduced in SM-Dicer KO aorta by approximately 95% and 99% respectively, suggesting an efficient knockdown of SMC miRNAs (Figure 1B and C). Some of the other miRNAs analyzed, as well as Dicer mRNA, may also be significantly expressed in fibroblasts and/or endothelial cells present in the aortic preparation where they would not be affected by the SM-specific Dicer KO, which would explain the higher expression levels compared to miR-145 in SM-Dicer KO. To further support this theory, deletion of dicer in smooth muscle did not alter the expression of the endothelial-specific miRNA, miR-126 (Figure 1C).

In order to establish if post-natal loss of Dicer causes any effect on the growth of juvenile mice, body weight was measured every 10 days for 9 weeks following tamoxifen or vehicle treatment (Figure 1D and Figure S1B). Some mice were sacrificed at 5 and 10 weeks and the tibial length was measured. Although there were no differences in tibial length (Figure 1D and Figure S1C), there was a slight, but significant reduction in body weight over time in SM-Dicer KO mice (Figure 1D). This may be related to our finding that the small intestines of SM-Dicer KO mice were distended and exhibited reduced peristaltic activity 10 weeks post-tamoxifen (Figure S1D). This effect was not apparent at earlier time points (5 weeks post tamoxifen) but a detailed analysis of visceral SMC function was not performed in the present study. Smooth muscle knockdown of Dicer was lethal at 12–14 weeks post-tamoxifen injection. The gastrointestinal phenotype of the SM-Dicer KO mice is the most likely cause of this lethality although this was not conclusively demonstrated.

### Conditional loss of Dicer in VSMC lowers blood pressure but does not change cardiac dimensions

**Figure 2:**
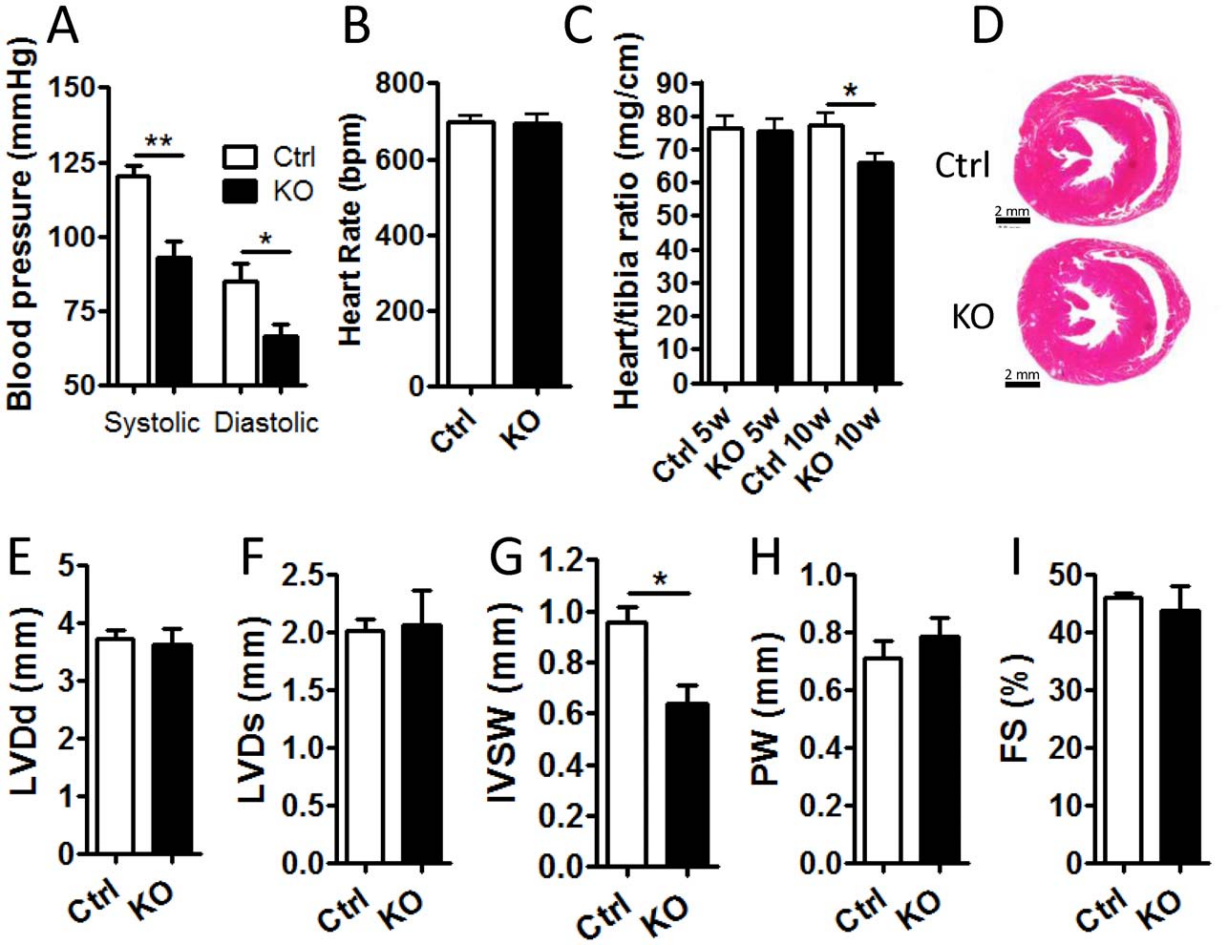
Blood pressure is reduced but cardiac function is maintained in SM-Dicer KO mice. (A) Blood pressure (systolic and diastolic)(A) and (B) heart rate was determined in conscious control (Ctrl) and SM-Dicer KO (KO) mice 6–8 weeks post-tamoxifen treatment, using tail cuff technique (n = 5 per group). (C) Cardiac size and function in WT and KO mice was investigated by measurement of wet weight vs. tibia length. (D) Representative H&E staining of Ctrl and KO hearts and (E-I) echocardiographic dimensions (LVDd, Left ventricular end-diastolic diameter; LVDs, Left ventricular end-diastolic diameter; IVSW, interventricular septal wall; PW, posterior wall; FS, fractional shortening). Data are expressed as mean+ SEM, with n = 3–4 for cardiac dimension studies.

One of the essential functions of VSMC is to maintain blood pressure by regulating the diameter of resistance arteries. Using the tail cuff technique, we analyzed systolic and diastolic blood pressure 6–8 weeks post-tamoxifen in conscious SM-Dicer KO and control mice. This time point was found to be optimal in order to obtain reliable blood pressure measurements while minimizing confounding effects due to compensatory mechanisms. As shown in Figure 2A, SM-Dicer KO mice exhibited a severe reduction in both systolic and diastolic blood pressures, while the heart rate was not altered (Figure 2B). Since these mice were trained extensively prior to the blood pressure measurement, blood pressure of control mice closely resembles the values obtained in freely moving mice using telemetry [15]. Interestingly, the drop in blood pressure was more severe in SM-Dicer KO mice compared to previously reported data on miR-143/145 KO mice, suggesting that additional miRNAs may regulate VSMC functions.

Analysis of cardiac size revealed a slightly reduced heart weight vs. tibia length in SM-Dicer KO animals 10 weeks post tamoxifen treatment (Figure 2C). This may be a consequence of decreased cardiac after-load due to reduced blood pressure considering that this effect was not observed at the earlier time point. Representative cross-sections of Dicer KO and control hearts are shown in Figure 2D where no apparent differences were observed in gross morphology between the two groups. Cardiac dimensions were monitored in age-matched anesthetized mice using echocardiography (Vevo 770). As shown in Figure 2E-I, SM-Dicer KO mice did not display any differences compared to controls in echocardiographic parameters including left ventricular end diastolic diameter (LVDd), left ventricular end systolic diameter (LVDs), posterior wall thickness (PW) and fractional shortening (FS), suggesting that the reduced blood pressure in SM-Dicer KO mice is not due to a defect in cardiac function. However, intraventricular septal wall thickness (IVSW, Figure 2G) was significantly decreased in SM-Dicer KO, in agreement with reduced ratio of heart wet weight/tibia length versus control mice. No significant effect of tamoxifen treatment on blood pressure or heart weight/tibia length ratio was observed in Cre negative mice (Figure S1E and S1F).

### Contractile function is markedly diminished in SM- Dicer KO mice

**Figure 3:**
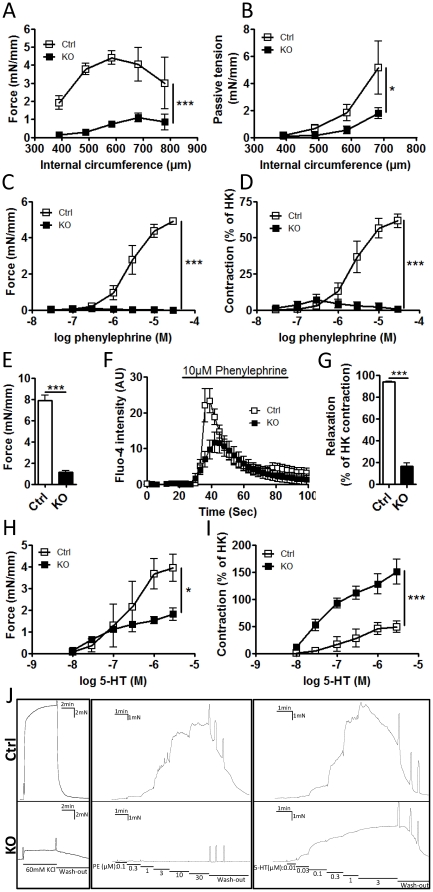
Severe impairment of contractile function in SM-Dicer KO arteries. (A) Contractile function of control and SM Dicer KO arteries was analyzed in a wire myograph, 10 weeks post Tamoxifen treatment. The active and passive circumference-tension relationships were analyzed in control (Ctrl) and SM-Dicer KO (KO) small mesenteric arteries. Active force in response to 80 mM KCl (HK) at different circumferences is summarized in A. In B, summarized data of the passive tension in nominally calcium free condition is shown. (C) Contractile responses to phenylephrine were measured in saphenous arteries and shown as absolute force or (D) force in relation to HK-induced responses. (E) Force in response to KCl in saphenous arteries is shown separately. (F) Relative phenylephrine-induced calcium influx in isolated aortic SMCs from Ctrl and KO mice were measured using Fluo-4 calcium indicator. (G) Represents summarized data of the remaining contractile tone following 1 min washout of the HK-induced contraction. Contractile responses to serotonin (5-HT) were measured in saphenous arteries and shown as absolute force (H) or force in relation to KCl-induced responses (I) (n = 3–4 mice for all experiments). An original trace recording from a representative experiment on saphenous artery is shown in J.

Blood pressure is in part regulated by the contractile function of vascular SMC in resistance arteries. Using wire myography, contractile function was examined in small mesenteric arteries and saphenous arteries from Ctrl and SM-Dicer KO mice 5 and 10 weeks post-tamoxifen. At 10 weeks post-tamoxifen, contractile force in response to depolarization by KCl (HK) was nearly abolished in small mesenteric arteries from SM-Dicer KO compared to Ctrl mice (Figure 3A). The circumference resulting in maximal force (Lmax) did not differ significantly between Ctrl mice and SM-Dicer KO (Lmax circumference; Ctrl, 658.8±46.7 vs. KO, 683±39.9). However, passive tension in calcium free conditions was significantly decreased in SM Dicer KO indicating an outward remodeling or altered compliance in these vessels (Figure 3B). Furthermore, the contractile response to the adrenergic α_1_-agonist phenylephrine was completely lost in SM-Dicer KO both when measured as absolute force and when related to HK-induced contraction (Figure 3C and 3D). Thus, the contractile response to phenylephrine was even further reduced than the HK-induced contraction in saphenous arteries shown in Figure 3E. Part of this effect could be due to a decreased phenylephrine-induced calcium influx in SM-Dicer KO, measured in isolated aortic SMCs and shown in Figure 3F. Following washout of HK-induced contractions we found that SM-Dicer KO arteries relaxed considerably slower than the control arteries. The remaining contractile tone 1 min after washout of KCl was thus determined and is summarized in Figure 3G. The cause of the reduced rate of relaxation in Dicer KO is not known but may be related to a perturbed myosin kinase and myosin phosphatase activity, which regulates the latch state in smooth muscle [31]. Another surprising finding, shown in Figure 3H and 3I, was that the contractile response to 5-HT was not as dramatically affected by SMC Dicer deletion as the phenylephrine- or HK-induced responses, indicating that loss of Dicer may differentiate between these signaling pathways. In support of this theory, calcium response to 5-HT in isolated SMCs did not differ between Ctrl and Dicer KO SMCs (Figure S2A). A representative trace recording of Ctrl and SM Dicer KO saphenous artery is shown in Figure 3J displaying the differences in HK-induced contraction, relaxation after washout and PE- and 5HT-induced contraction. The reduced ability of Dicer KO VSM to relax is apparent both following washout of HK-induced contraction and in the sustained contraction to 3 µM 5-HT which clearly differs from the transient contraction in Ctrl vessels.

At 5 weeks post-tamoxifen, contractile responses to HK were significantly reduced in SM-Dicer KO small mesenteric arteries but to a lesser extent compared to 10 weeks post-tamoxifen (Figure S2B). Lmax was significantly right-shifted at 5 weeks in the SM-Dicer KO mesenteric arteries ((Lmax circumference, Ctrl:605.1±19.5 vs. KO:748±32.5), which together with a decreased passive tension, shown in (Figure S2C), suggests an early outward remodeling of these vessels. In saphenous arteries, contractile responses to HK and phenylephrine were slightly reduced 5 weeks post-tamoxifen (Figure S2D-G). However, the response to 5-HT at this time point was dramatically increased both as absolute force and in relation to HK (Figure S2H and S2I). None of the contractile changes were due to unspecific effects of tamoxifen as shown using Tamoxifen and Vehicle-treated Cre-negative Dicer^floxed^ mice (Figure S2J and S2K).

### Deletion of Dicer in smooth muscle results in reduced aortic medial thickness

**Figure 4:**
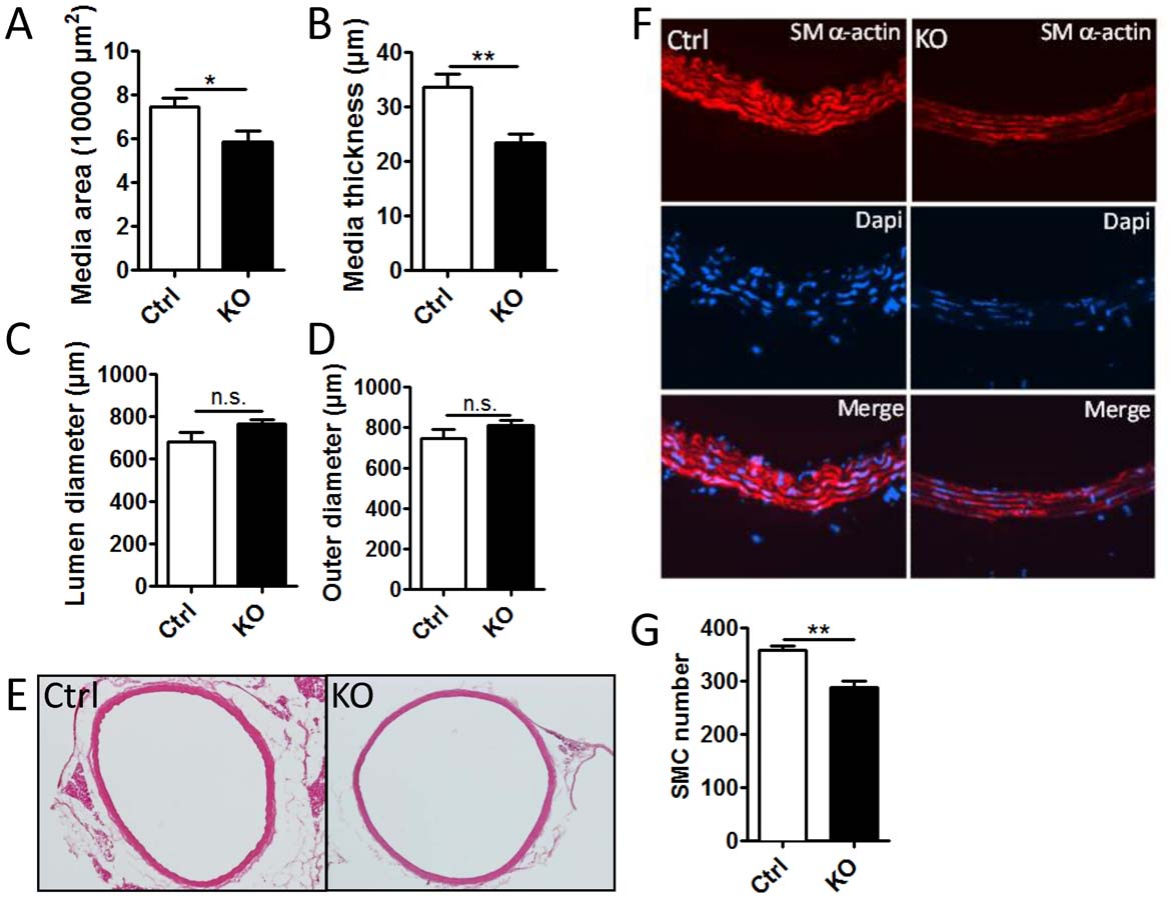
Morphological changes in the aorta of SM-Dicer KO mice. (A) Perfusion fixed and paraffin embedded sections of the aorta of control (Ctrl) and SM-Dicer KO (KO) mice were analyzed for morphological changes 10 weeks post tamoxifen treatment. Summarized data from 4–5 animals in shown in A-D. (E) Representative image of H&E stained Ctrl and KO aorta. (F) Representative images of Ctrl and KO aortas stained for SM-α-actin (red) and Dapi (blue). (G) Summarized data of SMC number in Ctrl and SM-Dicer KO aorta.

In our previous work we found that SM-Dicer KO in the embryonic vasculature resulted in an altered vascular geometry of E15.5 and E16.5 embryos with decreased wall thickness and medial area of the aorta [27]. This was associated with a reduced absolute number of SMCs in the media and a decreased expression of the proliferation marker Ki67. The amount of SMCs in the media is critical for the ability of vessels to contract and regulate blood pressure. Here, we analyzed vascular geometry of perfusion fixed, H&E stained aorta and saphenous artery from adult, control and SM-Dicer KO mice. Quantitative analysis of the aorta, shown in Figure 4A-D, revealed a decreased medial area and thickness but no change in diameters, indicating hypotrophy or loss of SMCs with concomitant compensatory remodeling. Representative images of control and SM-Dicer KO aortae are shown in figure 4E. In order to clarify if the decrease in media area in SM-Dicer KO aortae was due to loss of SMC or a decrease in SMC volume, we analyzed cell number in the media using Dapi nuclear staining. As shown in figure 4D and E, SMC numbers were significantly reduced in SM-Dicer KO mice suggesting that the decreased media area is due to a loss of SMCs. Furthermore, we observed a decreased SM α-actin staining of the aortic media of SM-Dicer KO mice, shown in Figure 4F, which indicates a reduced contractile differentiation in the remaining SMC of the aorta. Analysis of aortae from Cre-negative Dicer^floxed^ mice showed no effects of tamoxifen treatment (Figure S3A-D).

In saphenous artery, no differences in vessel dimensions or SMC number were observed between SM-Dicer KO and Ctrl mice, which suggest that the loss of contractile function in SM Dicer KO saphenous arteries is not due to a reduced smooth muscle mass (Figure S3F-K).

### Deletion of Dicer induces vessel bed-specific changes in medial SMC ultrastructure

**Figure 5:**
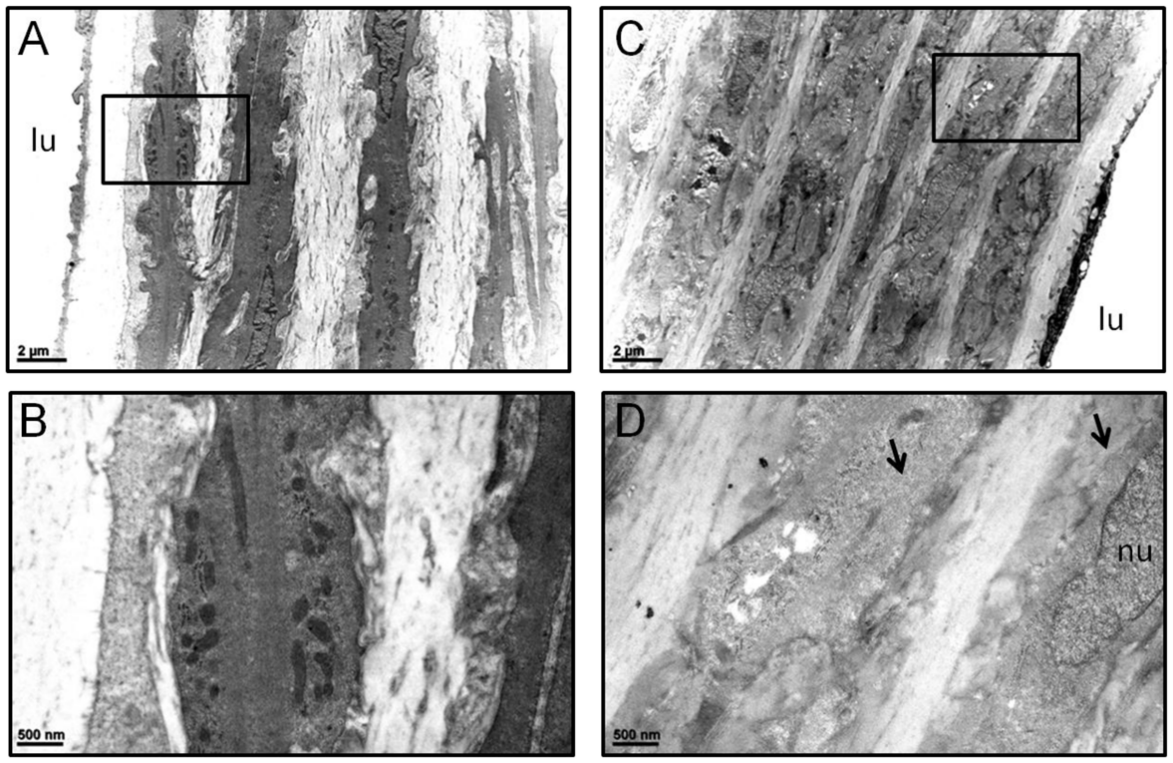
Altered smooth muscle cell ultrastructure in SM-Dicer KO aorta. Cross-sections of adult Ctrl (A,B) or SM-Dicer KO aorta (C,D). The rectangles in panels A and C represent magnified regions shown in panels B and D. Scale bars are indicated at bottom left of each panel. Arrows in panel D point to regions of SMC cytosol devoid of myofilaments. See Figure S4 for additional images of the Dicer KO ultrastructural phenotypes. lu, lumen; nu, nucleus.

We previously reported fragmented elastic lamellae and reduced myofilament content in embryonic aorta of SM-Dicer KO mice [27]. To determine whether inducible loss of Dicer in adult vessels elicits similar ultrastructural change, we performed, in a blinded manner, transmission electron microscopy (TEM) in both saphenous artery and thoracic aorta. We observed little change in the organization and myofilament content of medial SMC of saphenous artery in SM Dicer KO mice (data not shown), which correlates with the normal vascular geometry of this vessel. However, while the elastic lamellae and number of medial SMC layers (n = 5) appeared normal in control, aortic SMC, all three aortae from SM-Dicer KO mice showed attenuated myofilaments (Figure 5A-D and Figure S4A-C). In one Dicer null aorta, myelin figures could be seen throughout medial SMC suggesting cellular degeneration (Figure S4C) [32]. Taken together, results indicate vessel bed-specific changes in medial SMC ultrastructure with loss in normal Dicer expression.

### Loss of contractile differentiation in Dicer KO SMC

**Figure 6:**
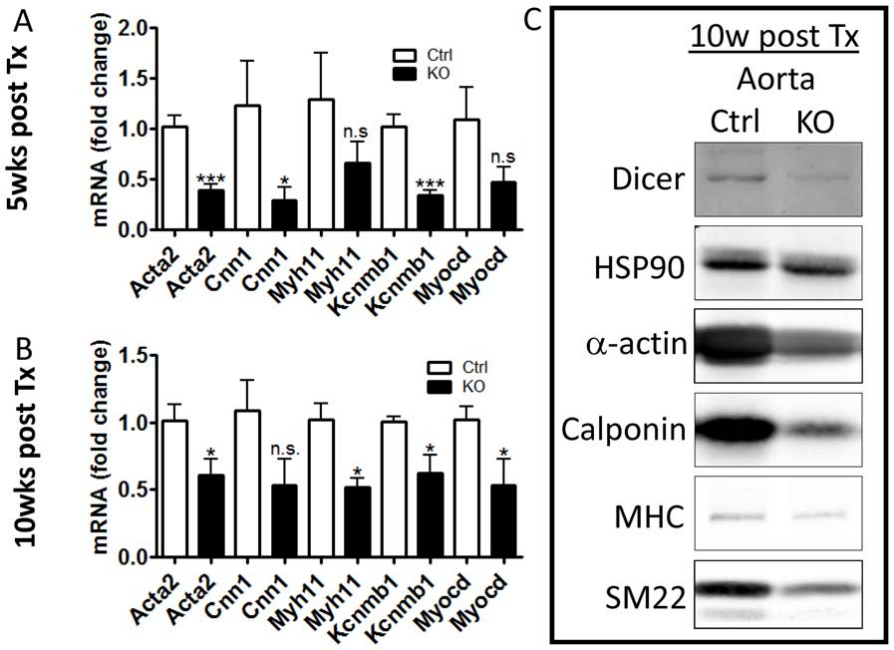
Impaired expression of markers of differentiation in SM-Dicer KO arteries. (A, B) Gene expression of several SMC markers were analyzed in control (Ctrl) and SM-Dicer KO aorta using RT-qPCR. Summarized data from 5 and 10 weeks post-tamoxifen are shown in A and B respectively (n = 3–6 vessels per group). (C) Representative Western blotting for Dicer and smooth muscle cell markers on extracts prepared from Ctrl and KO aorta, 10 weeks post-tamoxifen (n = 3). HSP90 was used as loading control.

The expression of contractile proteins is a requirement for contractile function of SMCs and is also used to determine the differentiated state of VSMC. Knockout of Dicer in embryonic SMCs result in the loss of SMC specific genes in umbilical artery [27]. As seen, in figure 6A and 6B, postnatal loss of Dicer in SMC of adult mice also result in reduced expression of contractile marker genes in the aorta including the genes encoding SM-α-actin (*Acta2*), calponin (*Cnn1*), SM-myosin heavy chain (*Myh11*) and β_1_-subunit of maxiK channel (*Kcnmb1*). All of these genes are regulated by the SRF co-factor myocardin [30], [33] and we found that myocardin expression was also reduced in SM-Dicer KO aorta which may explain the loss of SMC differentiation in SM-Dicer KO. The reduced expression of SMC markers in SM-Dicer KO aorta 10 weeks post tamoxifen was also confirmed at the protein level by Western blotting as shown in figure 6C. We have previously demonstrated a post-natal reduction of SMC specific genes and proteins in isolated aortic Dicer KO SMC *in vitro* [27]. Therefore it is likely that the reduced expression of SMC specific genes and proteins in SM-Dicer KO mice *in vivo* reflects a general reduction in SMC differentiation and not a loss of smooth muscle cells per se. Analysis of aortae from Cre-negative Dicer^floxed^ mice showed no significant effects of tamoxifen treatment (Figure S5).

## Discussion

Tissue-specific deletion of Dicer reveals the potential importance of miRNAs in the studied organ and provides a comparison for future studies of the role of specific miRNAs. The post-natal phenotype of conditional knock down of Dicer has previously been reported for cell types in the cardiovascular system including cardiomyocytes [18], endothelial cells [22] and hematopoetic stem cells [34], but not SMCs. In the present report we show that deletion of Dicer using a newly developed tamoxifen-inducible SMC specific mouse model results in widespread loss of miRNA expression and a dramatic reduction in blood pressure. Blood pressure is in part regulated by the contractile function of resistance arteries and we found nearly abolished contractile responses in these arteries in SM-Dicer KO mice. Furthermore we show that the contractile defect in SM-Dicer KO mice is associated with a loss of contractile differentiation of VSMCs as well as hypotrophic remodeling and loss of myofilaments in the aorta. These data support the idea that the terminally differentiated state of vascular smooth muscle requires miRNA synthesis and turnover.

The cause of the reduction in blood pressure in SM-Dicer KO mice is, most likely, primarily due to a reduced contractile tone in small resistance arteries as a consequence of a reduced expression of contractile proteins in the VSMCs. We did not find any significant difference in cardiac function between Ctrl and SM-Dicer KO mice, except for a slight decrease in wet weight and IVSW at 8–10 weeks, which is likely to be a consequence of a decreased cardiac after-load and not a cause of the decrease in blood pressure. The decrease in media thickness found in aortae of SM-Dicer KO mice may, hypothetically, also contribute to reduced blood pressure. However, since this effect was not observed in the smaller saphenous artery, remodeling of SM-Dicer KO arteries are not likely to be a major cause of reduced blood pressure. The compliance of the small arteries appears to be affected early in SM-Dicer KO mice since passive tension is right-shifted at both 5 and 10 weeks post tamoxifen. However, the importance of this effect in regulating blood pressure is uncertain.

Recently, we have shown that embryonic deletion of *Dicer* in VSMCs resulted in late lethality (E-15-E17) due to widespread hemorrhage [27]. This phenotype was associated with a decreased contractile function in umbilical arteries and a significantly reduced medial thickness of the aorta. In addition, contractile differentiation of the SMCs was reduced in SM-Dicer KO embryos. Interestingly, as shown in the present study, the embryonic phenotype is recapitulated when Dicer is deleted post-natally, which demonstrates that miRNAs are not only essential for SMC differentiation during the development of the vasculature but are also required in order to maintain the differentiated state of the SMC postnatally.

The importance of some specific miRNAs for SMC phenotypic modulation has been described previously and recently three separate reports were published presenting vascular phenotype of global miR-143/145 KO mice [13]–[15]. miR-143/145 are the first miRNAs suggested to be relatively specific for SMCs and play an important role for the regulation of SMC fate and maintenance of the contractile phenotype [6], [11]. In accordance, we found that over-expression of miR-145 rescued the loss of contractile differentiation in isolated Dicer KO SMCs [27]. Interestingly, although not lethal, the phenotype of miR-143/145 KO mouse closely resembles that of the inducible SM-Dicer KO mouse in several aspects. Firstly, systolic blood pressure of miR-145 and miR143/145 KO mice is reduced by approximately 15–20 mmHg [14], [15] while systolic blood pressure in SM-Dicer KO mice is reduced by 27.7 mmHg. In both mouse models this is associated with a decreased heart weight, likely secondary to a decreased after-load, while heart rate is unchanged. Secondly, miR-143/145 KO mice exhibit reduced contractile responses to KCl and phenylephrine [15] while these responses are nearly abolished in SM-Dicer KO mice 10 weeks post tamoxifen. Thirdly, both miR-143/145 KO mice and SM-Dicer KO mice have a decreased medial thickness [13]–[15] and a reduced SMC contractile differentiation [13], [15]. These similarities indicate that although miR-143/145 are not the essential miRNAs for SMC development they are important determinants of SMC differentiation and function *in vivo*. Alternatively, the milder phenotype of the constitutive miR-143/145 KO mice could partly be due to compensatory mechanisms.

As mentioned previously, we suggest that the decrease in blood pressure in SM-Dicer KO mice is likely due to a loss in SMC contractile differentiation. Herein, we found that deletion of Dicer in smooth muscle also resulted in reduced levels of myocardin mRNA. It has been suggested previously that myocardin expression is regulated by miR-145, either via direct binding to the 3'UTR of myocardin and translational activation [11] or via down-regulation of KLF5, a repressor of myocardin expression [6]. The loss of SMC contractile differentiation in SM-Dicer KO mice may thus be initially caused by a reduced miR-145 and myocardin expression. In addition, we previously reported that deletion of Dicer resulted in a dramatic loss of actin stress fibers, which was rescued by over-expression of miR-145 [27]. A similar loss of actin stress fibers was also observed in miR-145 KO SMC [14]. We also found that the potentiating effect of miR-145 on SMC contractile differentiation was abolished in Dicer KO SMCs pretreated with an inhibitor of actin polymerization [27]. Actin polymerization is known to be an important regulator of SMC contractile differentiation [35] and we have previously reported that actin dynamics is involved in stretch-induced contractile differentiation of vascular smooth muscle [36]–[39]. Thus, part of the decrease in SMC contractile differentiation in SM Dicer KO arteries may be due to depolymerization of actin filaments. This is further supported by our finding in the present study that aortae from SM-Dicer KO mice showed attenuated myofilaments by TEM analysis.

Our previous study on embryonic phenotypes of SM Dicer KO mice suggests that certain miRNAs are essential for SMC development but determining the identity of these miRNAs requires further investigation. The lethal phenotype of the tamoxifen-inducible SM-Dicer KO mice presented herein supports an essential role of miRNAs for vascular SMC function although the cause of death at >12–14 weeks post-tamoxifen is most likely related to a gastrointestinal and not a vascular phenotype. In conclusion, the present study provides the first insights into the global, post-natal importance of miRNAs in vascular SMC and we suggest that continual miRNA turnover regulates the levels of key genes in VSM that control contraction, remodeling and phenotypic modulation.

## Supporting Information

Figure S1 Tail DNA from Cre recombinase positive mice, wild type, homozygous or heterozygous for the floxed Dicer allele were genotyped before and two weeks after tamoxifen treatment. A representative agarose gel, which displays the presence of band representing the excised gene after tamoxifen treatment is shown in A. (B) analysis of body weight and tibia length (C) in Cre-negative (Cre-) mice treated with Vehicle (Veh) or Tamoxifen (Tx). (D) Representative image of control and KO small intestine, displaying the relaxed state of the intestinal smooth muscle in KO mice. (E) Heart/tibia ratio and blood pressure (D) in Cre-negative (Cre-) mice treated with Vehicle (Veh) or Tamoxifen (Tx).
(TIFF)

Figure S2 (A) Relative serotonin (5HT)-induced calcium influx in isolated Ctrl and KO isolated aortic SMCs were measured using Fluo-4 calcium indicator (A). (B-I) Contractile function of control and SM Dicer KO arteries 5 weeks post Tamoxifen treatment. (B) Active force of small mesenteric arteries in response to 80 mM KCl (HK). (C) Summarized data of the passive tension in nominally calcium free conditions. (D) Force in response to KCl in saphenous arteries. (E) Summarized data of the remaining contractile tone following 1 min washout of HK-induced contractions. Contractile responses to phenylephrine were measured in saphenous arteries and shown as absolute force (F) or force in relation to HK-induced responses (G). Contractile responses to serotonin (5-HT) were measured in saphenous arteries and shown as absolute force (H) or force in relation to KCl-induced responses (I). Contractile responses to phenylephrine in saphenous arteries from Cre-negative mice 10 weeks post Vehicle (Veh) or Tamoxifen (Tx) treatment shown as absolute force (J) or force in relation to HK-induced responses (K).
(TIFF)

Figure S3 (A-E) Morphological analysis and analysis of cell number of the aorta of Cre-negative (Cre-) mice, 10 weeks post Tamoxifen (Tx) or vehicle (Veh) treatment. (F-J) Perfusion fixed and paraffin embedded sections of the saphenous artery of control (Ctrl) and SM-Dicer KO (KO) mice were analyzed for morphological changes and cell number. (K) Representative image of H&E stained Ctrl and KO saphenous artery.
(TIFF)

Figure S4 Transmission electron micrographs of aortic SMCs of control and Dicer KO mice, 10 weeks post tamoxifen tratament. (A) Aortic SMC from control mouse. Note typical abundance of myofilaments in the cytosol of two adjacent medial SMC (black arrows) as well as peripheral dense plaques (arrowheads). (B) Aortic SMC from SM-Dicer KO mouse. Note the virtual absence of myofilaments in the cytosol of two adjacent medial SMC (black arrows). The nucleus (nu) of one SMC is labeled at left. (C) A medial SMC from a SM-Dicer KO aorta showing myelin figures that typify a cell undergoing degeneration.
(TIFF)

Figure S5 RT-qPCR analysis of selected mRNAs in the aorta of Cre-negative (Cre-) mice, 10 weeks post vehicle (Veh) or Tamoxifen (Tx) treatment.
(TIFF)
