# Supplementary figures and images for: Smooth Muscle miRNAs Are Critical for Post-Natal Regulation of Blood Pressure and Vascular Function
Source: PLoS One. 2011 Apr 22;6(4):e18869. doi: 10.1371/journal.pone.0018869 (PMC3081311; doi:10.1371/journal.pone.0018869)

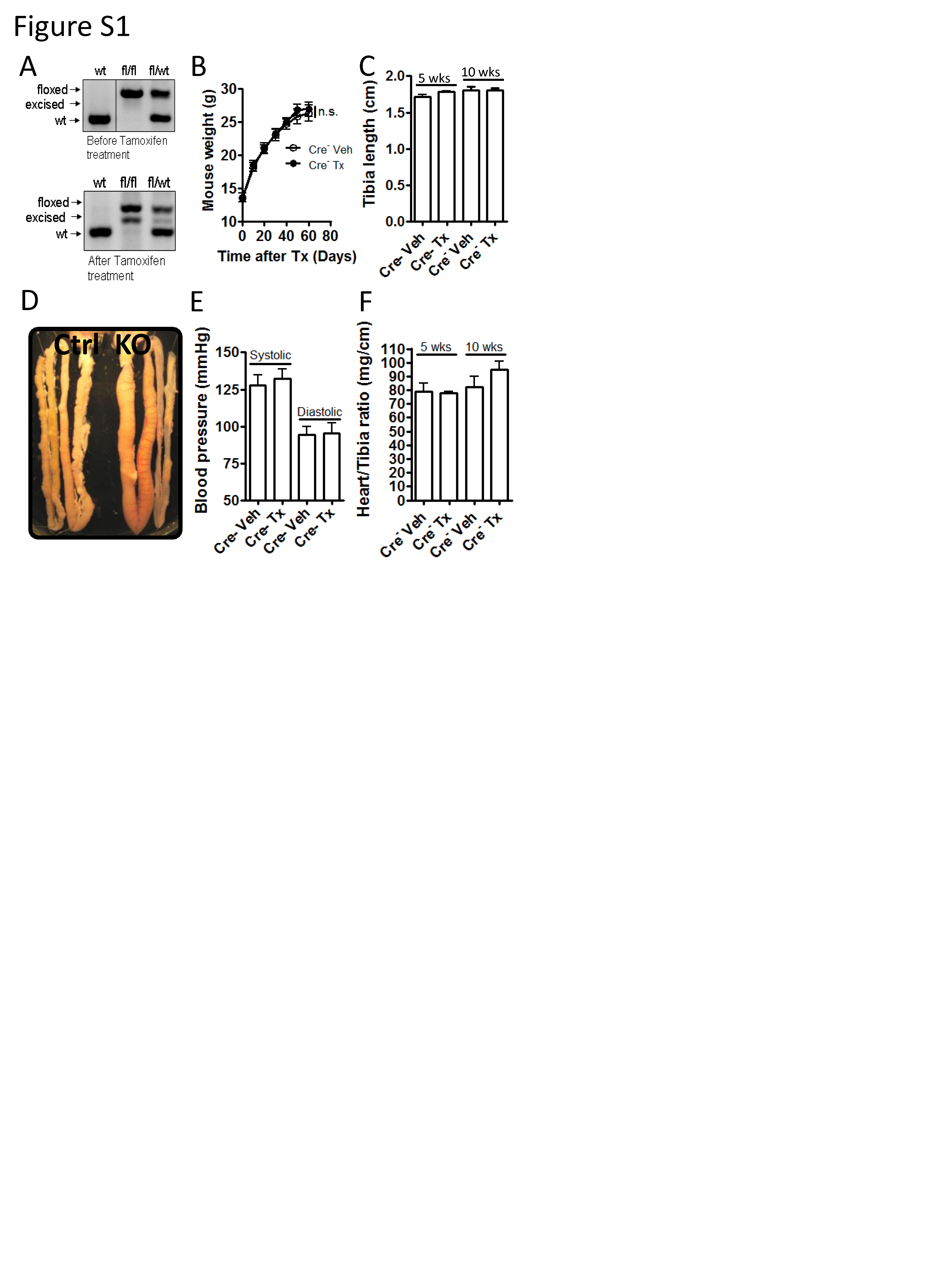

Supplement: Figure S1 — Tail DNA from Cre recombinase positive mice, wild type, homozygous or heterozygous for the floxed Dicer allele were genotyped before and two weeks after tamoxifen treatment. A representative agarose gel, which displays the presence of band representing the excised gene after tamoxifen treatment is shown in A. (B) analysis of body weight and tibia length (C) in Cre-negative (Cre-) mice treated with Vehicle (Veh) or Tamoxifen (Tx). (D) Representative image of control and KO small intestine, displaying the relaxed state of the intestinal smooth muscle in KO mice. (E) Heart/tibia ratio and blood pressure (D) in Cre-negative (Cre-) mice treated with Vehicle (Veh) or Tamoxifen (Tx). (TIFF) [file pone.0018869.s001.tiff]

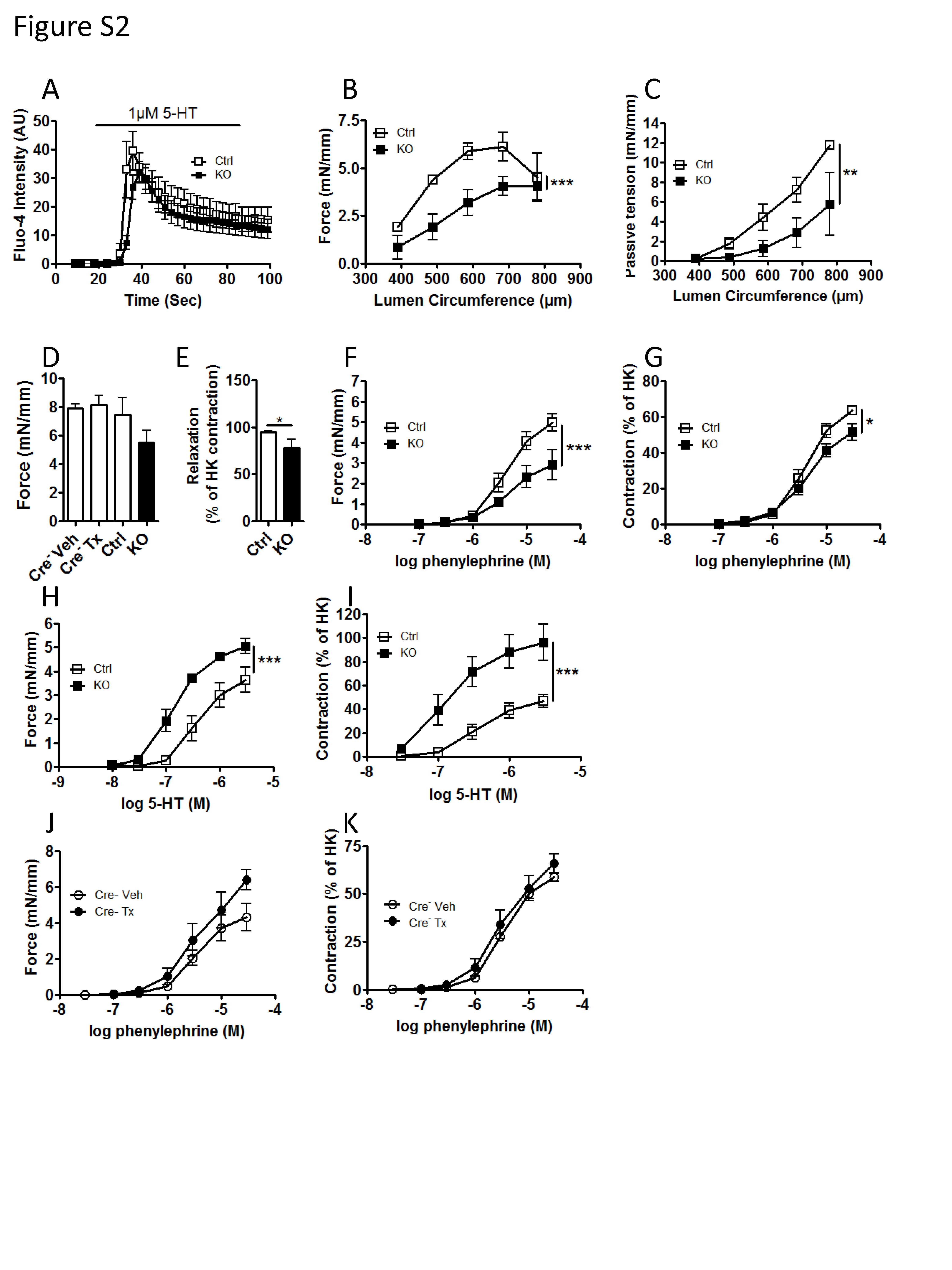

Supplement: Figure S2 — (A) Relative serotonin (5HT)-induced calcium influx in isolated Ctrl and KO isolated aortic SMCs were measured using Fluo-4 calcium indicator (A). (B-I) Contractile function of control and SM Dicer KO arteries 5 weeks post Tamoxifen treatment. (B) Active force of small mesenteric arteries in response to 80 mM KCl (HK). (C) Summarized data of the passive tension in nominally calcium free conditions. (D) Force in response to KCl in saphenous arteries. (E) Summarized data of the remaining contractile tone following 1 min washout of HK-induced contractions. Contractile responses to phenylephrine were measured in saphenous arteries and shown as absolute force (F) or force in relation to HK-induced responses (G). Contractile responses to serotonin (5-HT) were measured in saphenous arteries and shown as absolute force (H) or force in relation to KCl-induced responses (I). Contractile responses to phenylephrine in saphenous arteries from Cre-negative mice 10 weeks post Vehicle (Veh) or Tamoxifen (Tx) treatment shown as absolute force (J) or force in relation to HK-induced responses (K). (TIFF) [file pone.0018869.s002.tiff]

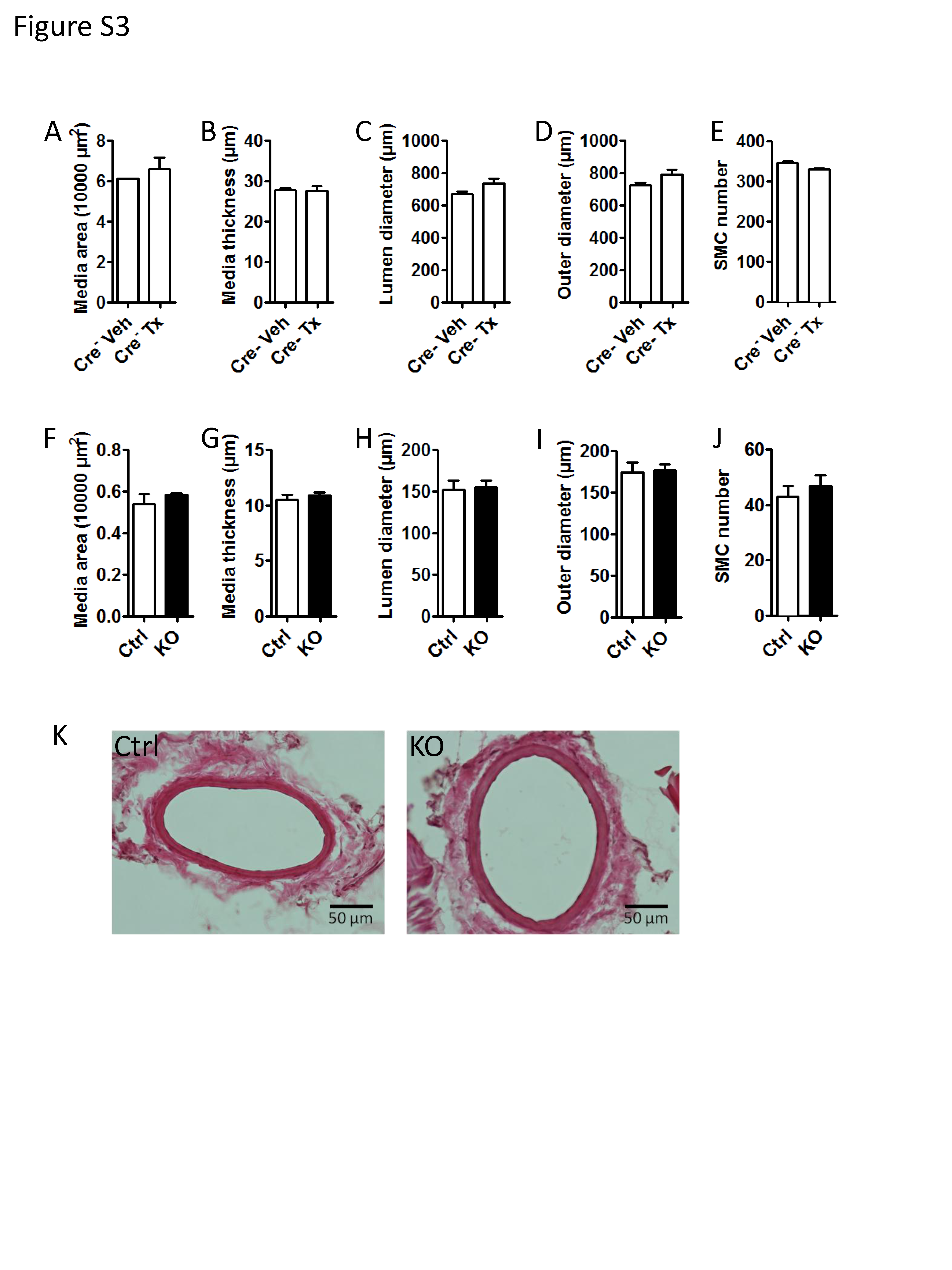

Supplement: Figure S3 — (A-E) Morphological analysis and analysis of cell number of the aorta of Cre-negative (Cre-) mice, 10 weeks post Tamoxifen (Tx) or vehicle (Veh) treatment. (F-J) Perfusion fixed and paraffin embedded sections of the saphenous artery of control (Ctrl) and SM-Dicer KO (KO) mice were analyzed for morphological changes and cell number. (K) Representative image of H&E stained Ctrl and KO saphenous artery. (TIFF) [file pone.0018869.s003.tiff]

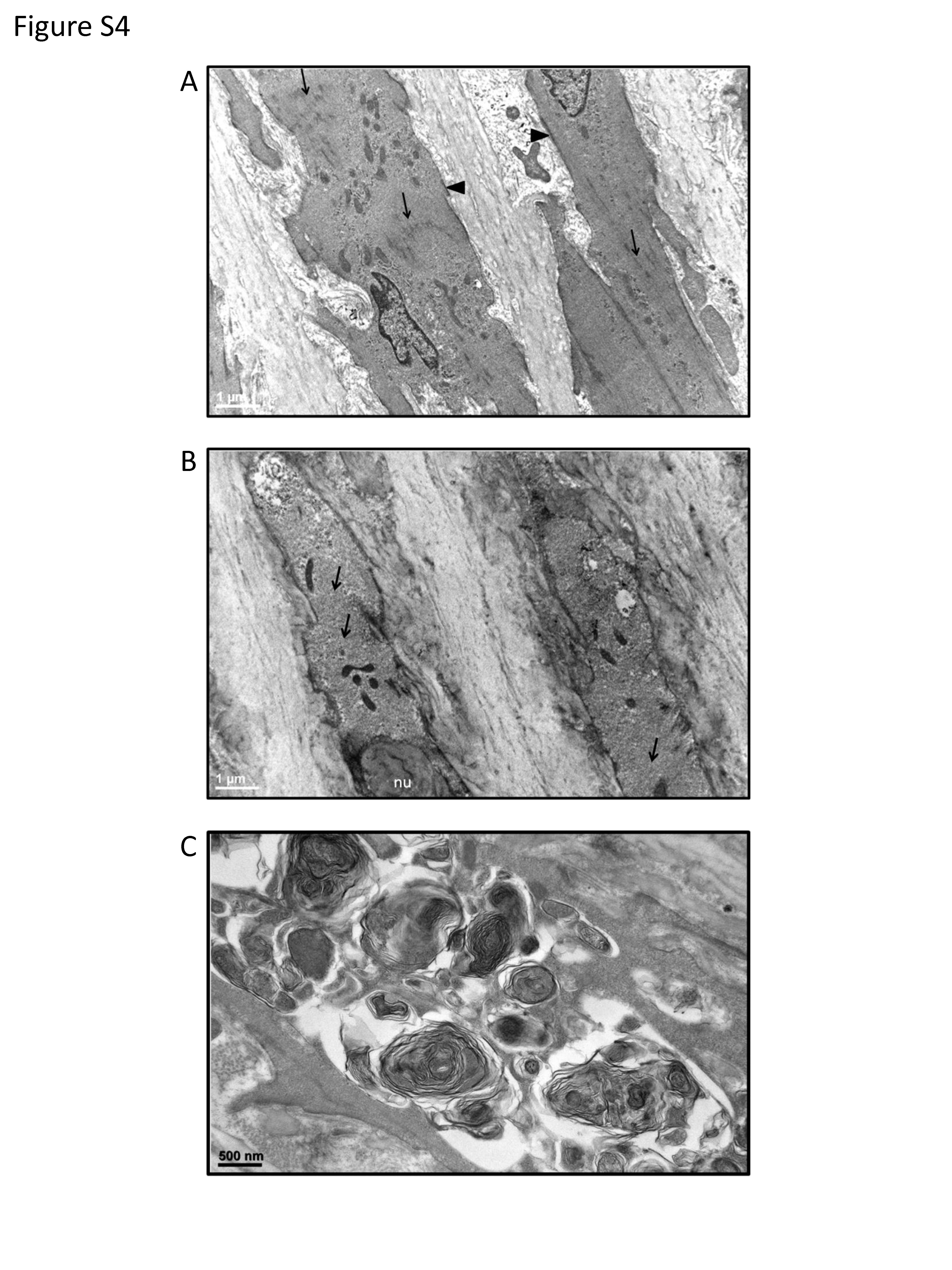

Supplement: Figure S4 — Transmission electron micrographs of aortic SMCs of control and Dicer KO mice, 10 weeks post tamoxifen tratament. (A) Aortic SMC from control mouse. Note typical abundance of myofilaments in the cytosol of two adjacent medial SMC (black arrows) as well as peripheral dense plaques (arrowheads). (B) Aortic SMC from SM-Dicer KO mouse. Note the virtual absence of myofilaments in the cytosol of two adjacent medial SMC (black arrows). The nucleus (nu) of one SMC is labeled at left. (C) A medial SMC from a SM-Dicer KO aorta showing myelin figures that typify a cell undergoing degeneration. (TIFF) [file pone.0018869.s004.tiff]

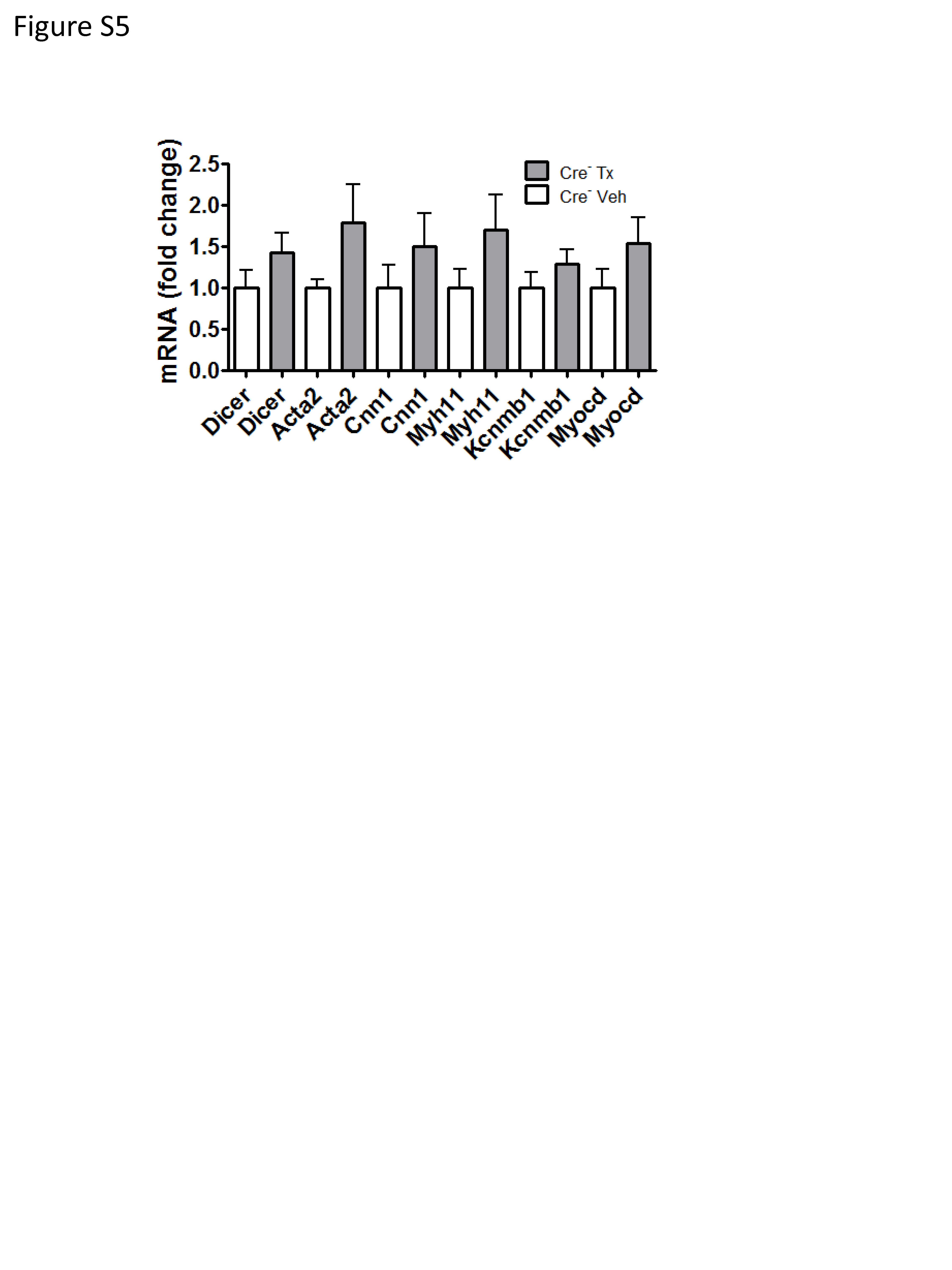

Supplement: Figure S5 — RT-qPCR analysis of selected mRNAs in the aorta of Cre-negative (Cre-) mice, 10 weeks post vehicle (Veh) or Tamoxifen (Tx) treatment. (TIFF) [file pone.0018869.s005.tiff]
